# Treatment policy change to dihydroartemisinin–piperaquine contributes to the reduction of adverse maternal and pregnancy outcomes

**DOI:** 10.1186/s12936-015-0794-0

**Published:** 2015-07-15

**Authors:** Jeanne Rini Poespoprodjo, Wendelina Fobia, Enny Kenangalem, Daniel A Lampah, Paulus Sugiarto, Emiliana Tjitra, Nicholas M Anstey, Richard N Price

**Affiliations:** Mimika District Health Authority, District Government Building, Jl. Cendrawasih, Timika, 99910 Papua Indonesia; Timika Malaria Research Programme, Papuan Health and Community Development Foundation, Jl. SP2-SP5, RSMM Area, Timika, 99910 Papua Indonesia; Department of Child Health, Faculty of Medicine, University Gadjah Mada, Jl. Kesehatan no 1, Sekip, Yogyakarta, 55284 Indonesia; Mitra Masyarakat Hospital, Jl. SP2-SP5-Charitas, Timika, 99910 Indonesia; National Institute of Health Research and Development, Ministry of Health, Jl. Percetakan Negara, Jakarta, 10560 Indonesia; Global and Tropical Health Division, Menzies School of Health Research, Charles Darwin University, Darwin, PO Box 41096, Casuarina, NT 0811 Australia; Division of Medicine, Royal Darwin Hospital, Darwin, NT 0810 Australia; Nuffield Department of Clinical Medicine, Centre for Tropical Medicine and Global Health, University of Oxford, Oxford, OX37LJ UK

**Keywords:** Impact, Dihydroartemisinin–piperaquine, Maternal malaria, Pregnancy outcome

## Abstract

**Background:**

In Papua, Indonesia, maternal malaria is prevalent, multidrug resistant and associated with adverse outcomes for mother and baby. In March 2006, anti-malarial policy was revised for the second and third trimester of pregnancy to dihydroartemisinin–piperaquine (DHP) for all species of malaria. This study presents the temporal analysis of adverse outcomes in pregnancy and early life following this policy change.

**Methods:**

From April 2004 to May 2010, a standardized questionnaire was used to collect information from all pregnant women admitted to the maternity ward. A physical examination was performed on all live birth newborns. The relative risks (RR) and the associated population attributable risks (PAR) of adverse outcomes in women with a history of malaria treatment to the risk in those without a history of malaria during the current pregnancy were examined to evaluate the temporal trends before and after DHP deployment.

**Results:**

Of 6,556 women enrolled with known pregnancy outcome, 1,018 (16%) reported prior anti-malarial treatment during their pregnancy. The proportion of women with malaria reporting treatment with DHP rose from 0% in 2004 to 64% (121/189) in 2010. In those with history of malaria during pregnancy, the increasing use of DHP was associated with a 54% fall in the proportion of maternal malaria at delivery and a 98% decrease in congenital malaria (from 7.1% prior to 0.1% after policy change). Overall policy change to more effective treatment was associated with an absolute 2% reduction of maternal severe anaemia and absolute 4.5% decrease in low birth weight babies.

**Conclusions:**

Introduction of highly effective treatment in pregnancy was associated with a reduction of maternal malaria at delivery and improved neonatal outcomes. Ensuring universal access to arteminisin combination therapy (ACT) in pregnancy in an area of multidrug resistance has potential to impact significantly on maternal and infant health.

**Electronic supplementary material:**

The online version of this article (doi:10.1186/s12936-015-0794-0) contains supplementary material, which is available to authorized users.

## Background

In Papua Province, Indonesia, *Plasmodium falciparum* and *Plasmodium vivax* infections in pregnancy are associated with adverse maternal and neonatal outcomes [1]. Malaria control in pregnancy in this region includes the use of long-lasting insecticide-treated nets (LLIN) and provision of early detection and prompt treatment with an effective anti-malarial drug. Prior to 2006 local guidelines recommended the use of sulfadoxine-pyrimethamine (SP) and chloroquine (CQ) for uncomplicated malaria. However clinical trials highlighted high levels of resistance to both compounds [2]. Whilst quinine is an alternative treatment in pregnancy, prolonged treatment regimens are required and these result in poor adherence. Unsupervised seven-day courses of quinine in non-pregnant women are associated with more than 50% recurrent infections within 28 days [2]. Recurrent febrile illness in pregnancy has major adverse effects on both mother and foetus including maternal anaemia, low birth weight, miscarriage and perinatal mortality [3–5].

In view of the extremely poor efficacy of standard anti-malarial treatment, the first-line treatment policy for uncomplicated malaria in Timika was changed in March 2006 to dihydroartemisinin–piperaquine (DHP) for all species of malaria and in the second and third trimesters of pregnancy [2, 6]. Patients with severe malaria were treated with intravenous artesunate.

In this population a review of more than 1,000 pregnant women, treated with DHP highlighted its safety profile and ability to reduce recurrent infections and malaria at delivery [3]. After more than 5 years of DHP deployment, the current study evaluates the temporal trends of maternal health and adverse pregnancy outcomes following policy change.

## Methods

### Study site

Until November 2008 the Rumah Sakit Mitra Masyarakat (RSMM) was the only hospital in the Mimika district, servicing a population of approximately 2,00,000. This lowland region is largely forested with a climate that varies little throughout the year. Rainfall in the lowlands is approximately 5,000 mm/year and varies little during the study period. Mean lowland temperatures are between 22 and 32°C. Vector control programme in this region covers approximately 40% of the population.

The area has unstable malaria transmission which an estimated annual incidence of 876 per 1,000 person years, divided 60:40 between *P. falciparum* and *P. vivax* infections [7]. Treatment failures in this area are extremely high, with 65% having recurrent failure on day 28 after CQ monotherapy for *P. vivax* and 48% on day 42 after CQ plus SP for *P. falciparum* [2].

### Study population

Each year there are approximately 3,000 pregnant women in Timika, of whom less than 40% attend antenatal care clinic [8]. Household surveys suggest that half of these women deliver at the RSMM hospital and the same proportion sleep under insecticide-treated nets (ITN) (Mr. Usman, District Health Malaria Officer, personal communication-2009). Intermittent presumptive treatment (IPT) for malaria is not available and HIV testing was not routinely carried out during the study period.

The ethnicity of the population includes lowland and highland Papuans, as well as non-Papuan ethnic groups attracted to the region by the local mine. In view of the high number of infections in non-immune patients, local protocols recommend that patients with parasitaemia detected by blood film examination irrespective of symptoms should be treated with anti-malarial drugs; this policy extends to pregnant women.

### Study design

Data were derived from a hospital-based malaria surveillance programme carried out between April 2004 and May 2010. Following informed consent all pregnant women and newborn infants admitted to the maternity ward at RSMM hospital were enrolled into the study. The attending physician or research clinician prospectively reviewed all admissions. A systematic interview and review of the clinical notes was used to obtain data on maternal characteristics, clinical condition (fever and signs of severity), history of possible malaria during the current pregnancy and its treatment, malaria treatment during hospitalization, and birth outcome (Additional file 1). All newborn infants were weighed and a full physical examination was conducted by a trained research nurse. Gestational age was estimated using the New Ballard Score [9].

Maternal thick and thin blood films were obtained to assess peripheral parasitaemia. Parasite counts were determined from the number of parasites per 200 white blood cells (WBC) on Giemsa-stained thick films and considered negative after review of 200 high-power fields. A thin smear was also examined to confirm parasite species and quantify parasitaemia if greater than 200 parasites were visible per 200 WBC. Haemoglobin concentration was determined by electronic coulter counter (Coulter JT™, USA). Malaria was defined as the presence of peripheral asexual parasitaemia irrespective of clinical signs or species. Maternal anaemia was defined as severe if the haemoglobin was less than 7 g/dl [10]. Neonatal adverse outcomes (low birth weight, preterm delivery and perinatal deaths) were defined according to WHO criteria [11]. On admission to hospital, fever was diagnosed if women gave a history of fever within the preceding 24 h or had an axillary temperature greater than 37.5°C.

Malaria was treated according to local protocols. Until March 2006 this was SP plus CQ for falciparum malaria and CQ alone for vivax malaria for the second and third trimesters of pregnancy. Quinine plus clindamycin was used for all species of malaria in the first trimester or when CQ and SP were not available. In March 2006 DHP became the recommended first-line treatment for uncomplicated malaria from any species of infection in the second and third trimester of pregnancy. Quinine and clindamycin were given in the first trimester. DHP (Artekin^®^, a fixed dose combination of 40 mg dihydroartemisinin and 320 mg piperaquine; Holley Pharmaceutical Co, PRC) was given according to the body weight with a target dose of 2–4 mg/kg of dihydroartemisinin and 16–32 mg/kg of piperaquine, once a day for 3 days. Following the publication of a multicentre severe malaria treatment trial in 2005, which included patients recruited from Timika, intravenous artesunate became the first-line treatment for severe malaria in pregnancy [12].

### Statistical analysis

Data were entered into EpiData 3.02 software (EpiData Association, Odense, Denmark) and statistical analysis done using SPSS vs17.0 (SPSS Inc, Chicago, USA). Categorical data were compared by χ^2^ with Yates’ correction or by Fisher’s exact test. To control for potential temporal changes in the patient demographics, the relative risk (RR) of adverse pregnancy outcomes were derived for women with history of malaria treatment compared to those without prior malaria during the same period. The period of analysis was divided into six blocks of 12 months starting from April 2004 to May 2010, with March 2006 as the time when treatment policy changed.

The proportion of women with malaria during pregnancy and its associated RR were used to calculate the population attributable risk (PAR) of prior malaria during pregnancy for each adverse outcome before and after policy change by using the following formula:

$${\text{PAR}}:\,\,\frac{{{\text{proportion of population exposed }} \times \, \left( {{\text{RR}} - 1} \right)}}{{ 1 { } + \, \left[ {{\text{proportion of population exposed }} \times \, \left( {{\text{RR}} - 1} \right)} \right]}}$$

The ratio of relative risks (RRR) between two relative risks and its significance was calculated according to the method of Altman and Bland [13]. The 95% confidence interval for PAR was calculated according to the delta method [14].

Previous studies have demonstrated that pregnant women with history of malaria in this region are at significantly greater risk of having malaria at delivery and other adverse pregnancy outcomes compared to women without any history of malaria [1]. The impact of anti-malarial policy change will be most apparent in women treated for malaria during pregnancy, the magnitude of this impact dependent upon the proportion of women with malaria receiving the new treatment regimen. Therefore, overall temporal trends of the adverse outcomes were presented after stratifying by those with prior malaria illness.

In order to control for confounding factors in measuring impact of malaria treatment policy change to maternal and pregnancy outcomes, this study examined the seasonal variation in Mimika as well as the annual proportion of primigravidae and pregnant women aged ≤16 years old [1].

### Ethical approval

Ethical approval for this study was obtained from the ethics committees of the National Institute of Health Research and Development, Ministry of Health, Indonesia (KS.02.01.2.1.3431) and Menzies School of Health Research, Darwin, Australia (04/47).

## Results

**Figure 1:**
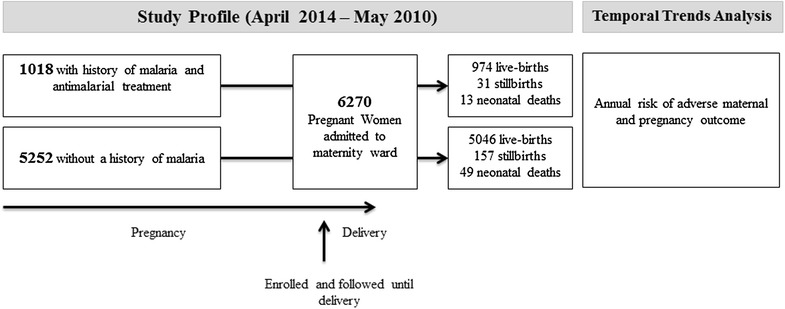
Study and analysis profile.

In total, 7,744 pregnant women were enrolled in the study. Of the 6,556 (85%) women with known pregnancy outcomes, 286 were excluded (283 with miscarriage and three women with missing data). Of the remaining 6,270 pregnant women, 1,018 (16%) had a history of malaria treatment during the current pregnancy (Figure 1).

**Figure 2:**
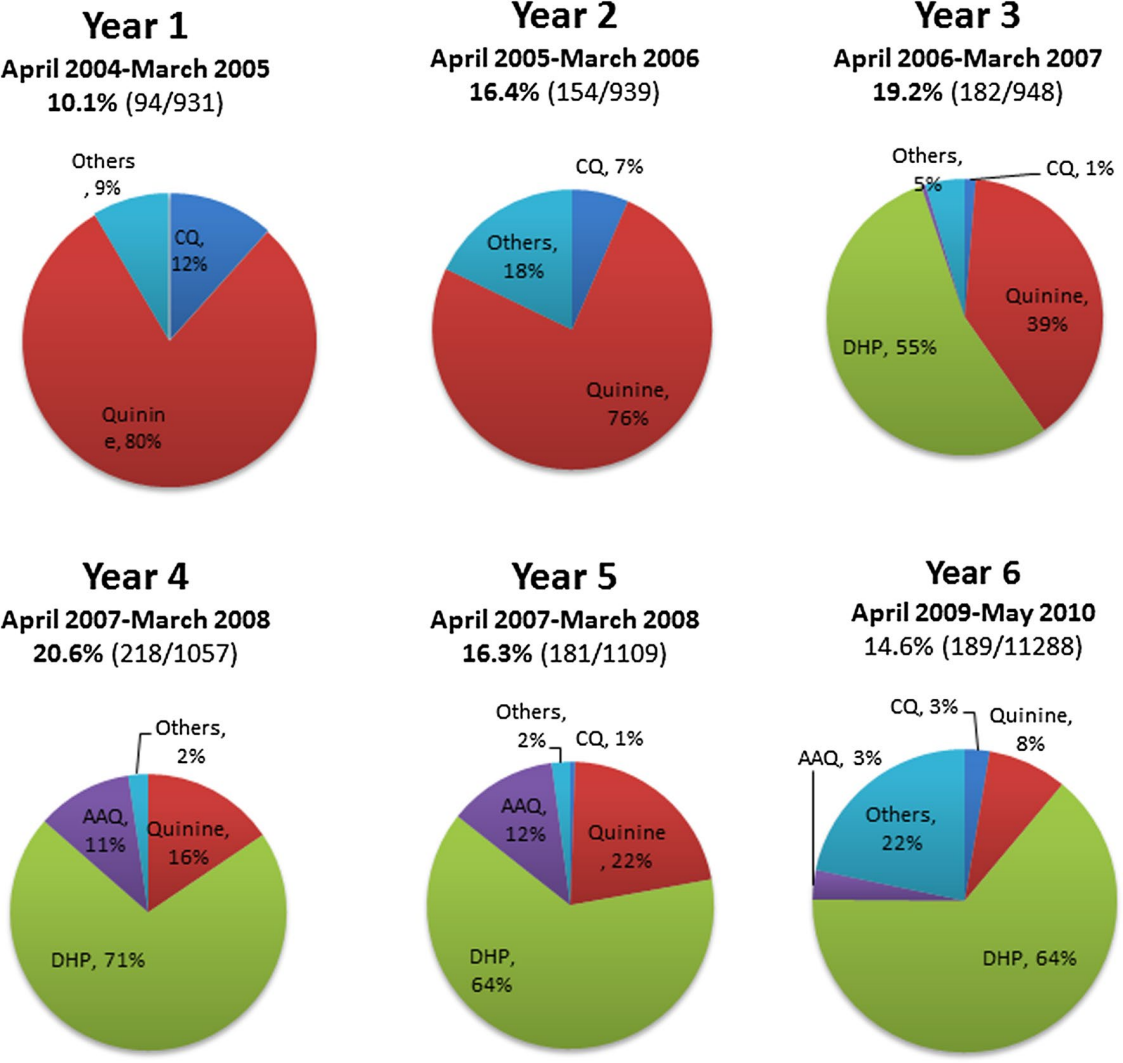
Prior anti-malarial treatment during pregnancy in women delivering. *Numbers* above the *pie charts* are proportion of pregnant women with history of malaria treatment in that year.

Over the duration of the study, the proportion of women delivering with a history of malaria and malaria treatment during pregnancy increased initially from 10% (94/931) in 2004 to 21% (218/1,057) in year 4 (April 2007–March 2008), p < 0.001, before decreasing to 14.6% (189/1,288) in year 6 (Figure 2). In women with a history of malaria treatment, the proportion receiving DHP rose from zero prior to policy change to 64% by the end of the study (Figure 2). The RR and the associated PAR of the adverse outcomes stratified by history of antenatal malaria treatment prior to and following treatment policy change are presented in Table 1.

**Table 1:** Adverse maternal and pregnancy outcomes before and after treatment policy change

Adverse outcomes	Women with history of malaria during pregnancy	Women without history of malaria during pregnancy	Relative risks (95% CI)	P value	Population attributable risks % (95% CI)
March 2004–March 2006: before treatment policy change					
Maternal malaria: delivery, n/valid cases (%)	142/246 (57.7)	199/1,592 (12.5)	4.6 (3.9–5.5)	<0.001	34.5 (29.3–39.8)
Primigravidae	56/91 (61.5)	70/490 (14.3)	4.3 (3.3–5.6)	<0.001	30.5 (22.1–38.9)
Multigravidae	86/155 (55.5)	129/1,099 (11.7)	4.7 (3.8–5.8)	<0.001	33.1 (26.8–39.3)
Maternal severe anaemia, n/valid cases (%)	40/241 (16.6)	152/1,588 (9.6)	1.7 (1.3–2.4)	<0.001	8.8 (4.4–13.4)
Low birth weight, n/valid cases (%)	50/244 (20)	215/1,608 (13.4)	1.5 (1.2–2.0)	0.003	6.6 (3.2–10.0)
Preterm delivery, n/valid cases (%)	27/225 (12)	110/1,503 (7.3)	1.6 (1.1–2.4)	0.015	7.8 (2.7–12.9)
Perinatal deaths, n/valid cases (%)	11/248 (4.4)	41/1,619 (2.5)	1.75 (0.9–3.2)	0.1	9.1 (0.1–18)
Congenital malaria, n/valid cases (%)	14/195 (7.1)	14/706 (1.9)	3.8 (1.8–8.2)	<0.001	25.7 (3.5–48)
April 2006–May 2010: After treatment policy change					
Maternal malaria: delivery, n/valid cases (%)	201/770 (26.1)	508/3,632 (13.9)	1.9 (1.6–2.2)	<0.001	13 (10.3–15.9)
Primigravidae	81/265 (30.6)	185/1,112 (16.6)	1.8 (1.5–2.3)	<0.001	12.7 (7.9–17.5)
Multigravidae	120/505 (23.8)	324/2,515 (12.9)	1.8 (1.5–2.2)	<0.001	12.8 (9.3–16.3)
Maternal severe anaemia, n/valid cases (%)	70/738 (9.5)	237/3,510 (6.8)	1.4 (1.1–1.8)	0.01	6.6 (3.2–10.1)
Low birth weight, n/valid cases (%)	123/764 (16.1)	521/3,615 (14.4)	1.1 (0.9–1.3)	0.23	2 (0.1–3.9)
Preterm delivery, n/valid cases (%)	121/740 (16.4)	462/3,472 (13.3)	1.2 (1.0–1.5)	0.03	3.8 (1.8–5.8)
Perinatal deaths, n/valid cases (%)	27/770 (3.5)	165/3,632 (4.5)	0.8 (0.5–1.2)	0.21	n/a
Congenital malaria, n/valid cases (%)	1/743 (0.1)	6/3,543 (0.2)	0.8 (0.1–6.6)	0.21	n/a

Valid cases refer to presence of variables required for the analysis.

**Figure 3:**
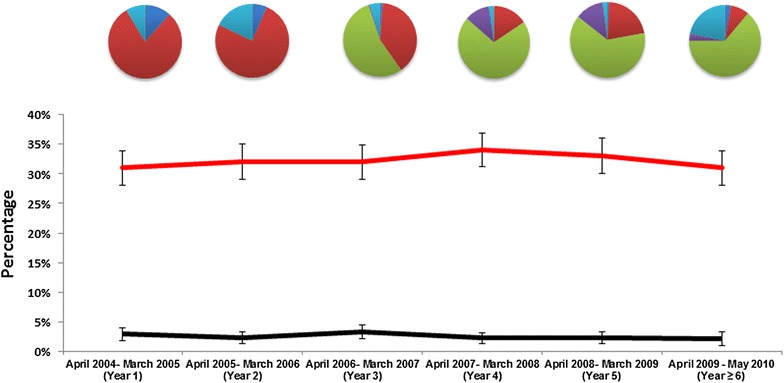
The yearly proportion of primigravidae (*red line*) and pregnant women age ≤16 years old (*black line*). The *pie chart* above the figures denote the proportions of women with prior anti-malarial exposure: Quinine: *red*, DHP: *light green*, CQ ± SP: *light blue*, AAQ: *purple*, others: *dark blue.*

There was no difference in the proportion of pregnant women who were primigravidae and aged less than 16 years old during the observation period (Figure 3). Women with a history of malaria treatment were more likely to be younger (age ≤ 16 years old) and first-time mothers compared to those without history of malaria illness: odds ratio of 1.5 (95% CI 1.02–2.2, p = 0.039) and 1.2 (95% CI 1.1–1.4, p = 0.008), respectively.

### Maternal adverse outcomes

**Figure 4:**
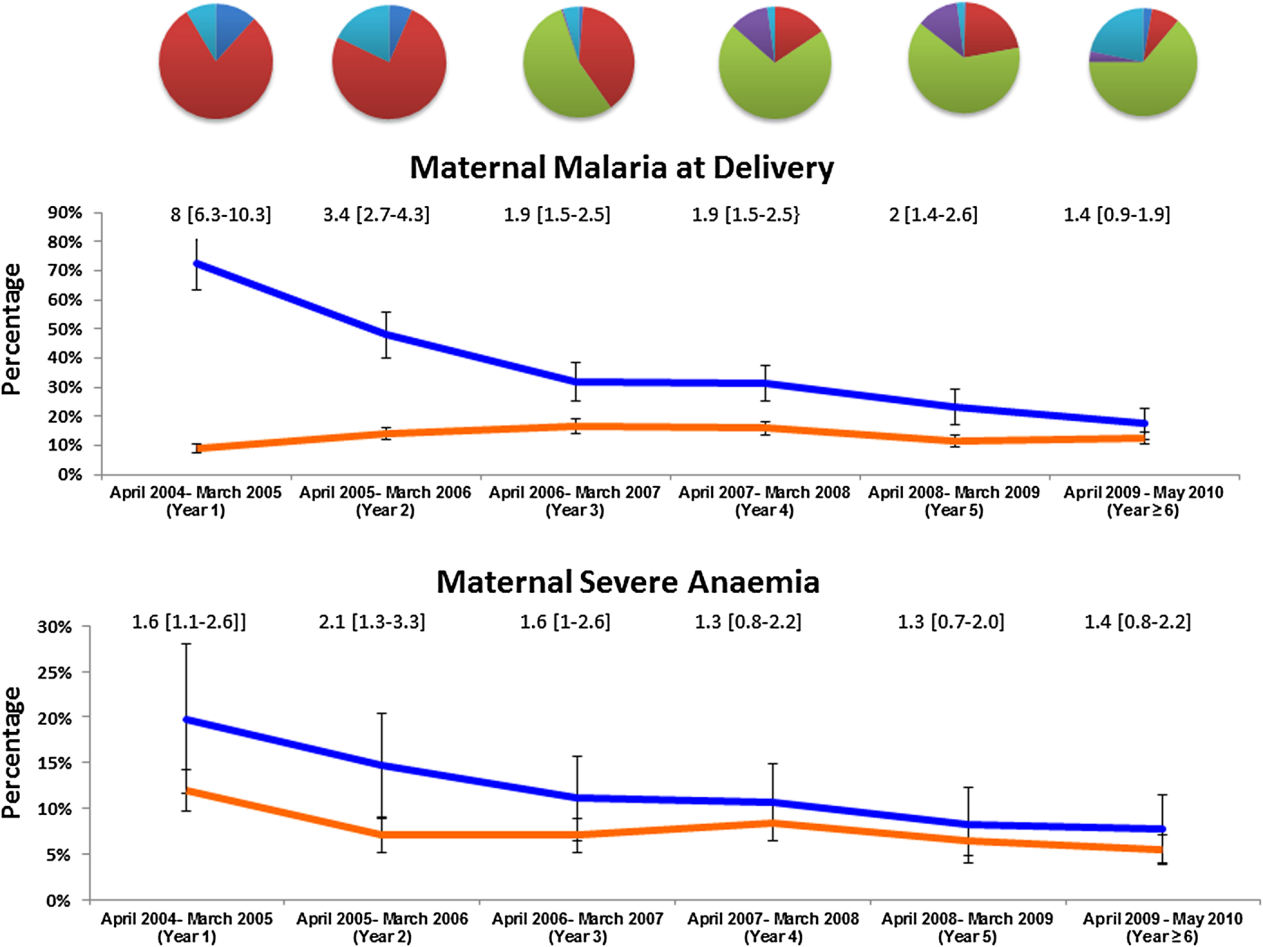
Maternal adverse outcomes in women with history of malaria treatment during pregnancy (*blue line*) and in those without history of malaria treatment (*orange line*). The *pie chart* above the figures denote the proportions of women with prior anti-malarial exposure: Quinine: *red*, DHP: *light green*, CQ ± SP: *light blue*, AAQ: *purple*, Others: *dark blue*. *Numbers* above the lines are RR (95% CI). *Black arrow* marks the start of policy change.

There was a significant reduction in the prevalence of malaria at delivery following the introduction of DHP. In women with a history of prior malaria treatment, the proportion with malaria at delivery declined from 57% (142/248) before DHP deployment to 26% (201/770) after policy change, p < 0.001 (Figure 4).

A similar pattern was noted in the prevalence of severe maternal anaemia (Figure 4), the risk of severe anaemia declining steadily over the 5 years of the study in both women with and without a history of malaria treatment. The proportion of women with severe anaemia declined from 10.5% (192/1,829) before DHP deployment to 7.2% (307/4,248) after policy change, p < 0.001 (Figure 4). The RR associated with history of malaria during pregnancy changing from 1.7 (95% CI 1.3–2.4) prior to policy change to 1.4 (95% CI 1.1–1.8) after policy change [RRR: 2.6 (95% CI 2.1–3.3), p < 0.001]. This equated to a change in PAR of 9% (95% CI 4–13%) pre- and 7% (95% CI 3–10%) post-policy change.

### Adverse outcomes of the newborn

**Figure 5:**
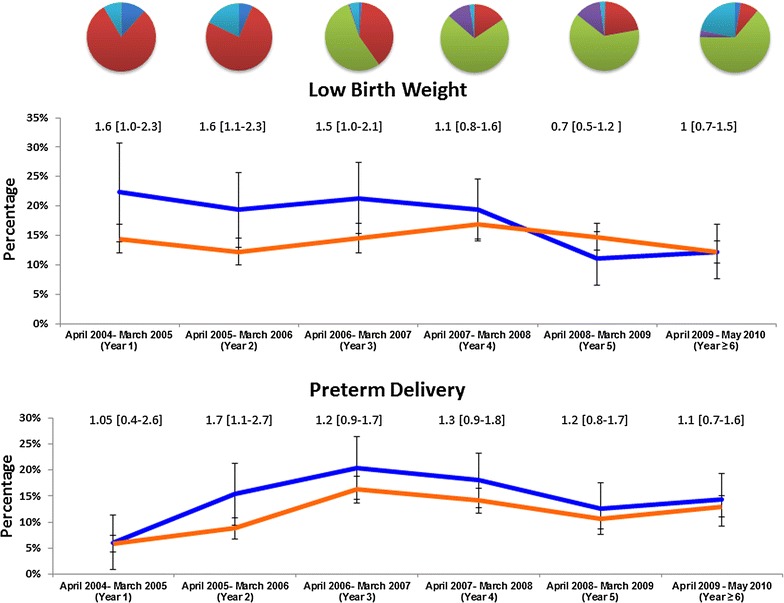
Adverse pregnancy outcomes in pregnant women with history of malaria treatment (*blue line*) and in those without history of malaria treatment (*orange line*). The *pie chart* above the figures denote the proportions of women with prior anti-malarial exposure: Quinine: *red*, DHP: *light green*, CQ ± SP: *light blue*, AAQ: *purple*, Others: *dark blue*. *Numbers* above the lines are RR (95% CI). *Black arrow* marks the start of policy change.

The proportion of low birth weight (LBW) babies born of mothers without a history of malaria was 14% (736/5,223) and did not change significantly over the study period. However in mothers with prior malaria treatment the prevalence of LBW fell significantly from 20% (50/244) in the first 2 years of the study, to 12% (23/188) in year 6, p = 0.03 (Figure 5). Prior to DHP deployment, the risk of LBW in women with a history of malaria treatment was 1.5 (95% CI 1.2–2.0) compared to 1.1 (95% CI 1–1.3) after policy change [RRR: 1.4 (95% CI 1–1.9), p = 0.05]. The PAR of LBW associated with prior malaria treatment decreased from 6.6% (95% CI 3–10%) to 2% (95% CI 0.1–4%) following treatment policy change.

The prevalence of preterm delivery rose significantly prior to DHP deployment, but was followed by a decline after year 3 (Figure 5). Although these trends were apparent in both women with and without a history of malaria, the change was greater in women with a history of malaria. The RR of prematurity associated with prior history of malaria fell from 1.6 [95% CI 1.1–2.4] to 1.2 [95% CI 1–1.5] after policy change [RRR: 1.3 (95% CI 0.9–2.1), p = 0.199], the PAR decreasing from 7.8% (95% CI 3–13%) to 3.8% (95% CI 2–6%).

Over the study period perinatal death was recorded in 250 (4%) of the 6,273 women delivering. There was no significant change in the risk of perinatal death associated with prior anti-malarial treatment; overall approximately 4.3% (44/1,018) in women with history of malaria treatment versus 4% (206/5,252) in those women without prior anti-malarial exposure, p = 0.54.

After March 2006, the relative risk of maternal to foetal malaria transmission associated with history of malaria treatment declined from 3.8 (95% CI 1.8–8.2) to 0.8 (95% CI 0.1–6.6), p = 0.17.

## Discussion

The local Ministry of Health revised the anti-malarial policy in Timika in March 2006, and since this time the public health care sector has knowingly treated more than 2,900 pregnant women with DHP. At the sentinel site at the RSMM the clinical research team have documented DHP administration to 765 women, and recorded deliveries in 847 women known to have been exposed to DHP during pregnancy [3]. In the current analysis this surveillance network is used to evaluate the impact of the policy change on adverse pregnancy and foetal outcomes at the hospital maternity ward.

This study documented an initial rise in the number of women delivering with history of malaria and malaria treatment during pregnancy from 11% (79/693) in 2004 to 20% (208/1,026) in 2007. During this period community and entomology unpublished studies conducted in parallel also demonstrated a paradoxical transient increase in the number of malaria cases in the general population. This increase in malaria coincided with an El Nino year, with a rise in vector numbers and malaria cases noted across Indonesia in the national statistics. Unpublished evidence also suggests additional effects from a shift in treatment-seeking behaviour resulting in improved surveillance following DHP introduction. Despite the increased number of pregnant women presenting with malaria after the introduction of DHP, no corresponding increased risk in adverse outcomes was observed.

The interpretation of temporal trends of malaria-related morbidity is complex. Treatment, patient demographics and health-seeking behaviour of women presenting to the antenatal clinics vary over time. The preliminary analyses of the temporal data in this study were based on simple before-and-after comparisons and did not take into account the trajectory of trends nor the likely autocorrelation of the data. For these reasons, interpretation of the study findings requires caution.

In women with antenatal malaria, the introduction of DHP was correlated with a 50% reduction in the risk of peripheral parasitaemia at delivery from 57 to 26%. The RR of malaria at delivery associated with prior treatment was 4.6 (95% CI 3.9–5.5) before policy change to 1.9 (95% CI 1.6–2.2] after DHP deployment, p < 0.001. The PAR of malaria at delivery was 34% (95% CI 29–40%) before March 2006 and 13% (95% CI 10–16%) thereafter. This figure shows that 21% of maternal malaria at delivery could be prevented by treatment policy change. The reduction in the maternal malaria prevalence associated with policy change was similar between primi and multigravidae women.

The fall in the proportion of women with peripheral parasitaemia at delivery was associated with a dramatic reduction in the risk of congenital malaria from 3 to 0.2% [15]. Indeed, there have been no more congenital cases of malaria since October 2008.

There was a significant reduction in the RR of LBW and a non-significant fall in severe maternal anaemia associated with antenatal malaria. The corresponding PAR fell from 7 to 2% and 9 to 7%, respectively. LBW and severe maternal anaemia are associated with frequent malaria episodes and the timing of infections [16], and it is likely that the reduction in these outcomes is due in part at least, to a reduction in the risk of recurrent malaria. The effect however was modest and this may reflect the limited access of mothers in this region to effective anti-malarial treatment. Inadequate quality of antenatal care would also compromise the pregnancy and health outcomes of pregnant women with relapsing *P. vivax* infections, which accounts for a third of maternal malaria in this area [1]. The introduction of DHP was associated with a slight reduction in the risk of preterm delivery, however an unexplained increased in the proportion of this adverse outcome before policy change confounds the interpretation of this observation.

This study has several limitations. Firstly, it was not possible to evaluate the overall programme impact in the community since this study used hospital-based surveillance. Although 76% of women came to the hospital for delivery, selection bias and treatment-seeking behaviour remain potential confounders of the analysis. However, the condition was controlled by comparing the RR of adverse outcomes in women with and without a history of malaria treatment.

Secondly, a history of symptomatic malaria and anti-malarial treatment during pregnancy was obtained by interview, a method that is subjected to recall bias. However, pregnant women in this region are very familiar with malaria symptoms and alternative anti-malarial drugs. A systematic interview method and review of the clinical notes revealed that the history of malaria treatment could be cross-checked in almost 50% of women with excellent concordance between reported drug history and that documented from the records. Unfortunately, the interview method did not gather information on possible asymptomatic and untreated malaria in pregnant women, and this is likely to have underestimated the effectiveness of treatment policy change.

The study highlights the potential benefits of improving maternal and pregnancy outcomes in women through the introduction of more efficacious anti-malarial treatment regimens. However, the wider impact on health targets will require improvements in case management and prevention programmes of malaria in pregnancy as well as implementation of strategies to ensure the delivery of universal coverage [17, 18]. In Papua, even though malaria control programmes in pregnancy focus on providing early diagnosis and prompt treatment, in reality this capacity is available in less than half of the local health facilities with limited public health impact [7]. The challenges in the prevention of malaria are similar, highlighted by an ITN distribution programme to pregnant women in Timika district, initiated in 2007, which has achieved only 50% coverage.

## Conclusions

The deployment of DHP for malaria in the second and third trimesters of pregnancy was associated with a significant decrease in adverse maternal and infant outcomes. This study paves the way for scaling up both treatment and prevention programmes using arteminisin combination therapy. The latter will require identifying the most effective method of service delivery and translating research into practice (Additional file 1).

## Additional file

Additional file 1: Pregnant women form.
